# When the Infection Clears but Inflammation Persists: A Case of Chlamydia-Associated Reactive Arthritis Requiring Disease-Modifying Antirheumatic Drug (DMARD) Therapy

**DOI:** 10.7759/cureus.101878

**Published:** 2026-01-19

**Authors:** Zaineb Khawar, Ifunanyachukwu Umeozor, Arsany Anis

**Affiliations:** 1 Internal Medicine, Saint Michael's Medical Center, Newark, USA; 2 Internal Medicine, St. George's University School of Medicine, Newark, USA; 3 Rheumatology, Jersey City Medical Center, Jersey City, USA

**Keywords:** chlamydia infection, dmard (disease modifying anti rheumatic drugs), oligo-arthritis, reactive arthritis, seronegative inflammatory arthritis

## Abstract

Reactive arthritis (ReA) can be a persistent and debilitating form of arthritis that is a consequence of a gastrointestinal or genitourinary infection. Although most cases self-resolve, a small subset of cases may persist and require an escalation of care to manage their symptoms. We present a case of recurrent ReA induced by *Chlamydia trachomatis *infection in a young male patient who required a stepwise escalation of his medical management. This case outlines a 32-year-old man followed over six months from his diagnosis. Initially, the patient presented with persistent joint pain in the lower extremities, elevated inflammatory markers, a positive HLA-B27 antigen, and a positive *Chlamydia* test. He was treated for his initial symptomatology with antibiotics and systemic steroids, and later, methotrexate was added, with clinical improvement. However, several months later, the patient relapsed and required the initiation of a biologic disease-modifying antirheumatic drug (DMARD). This case is an opportunity to follow a case of *C. trachomatis-*induced ReA, and through a literature review, it details the considerations clinicians must take into account to manage their patients’ symptomatology.

## Introduction

Reactive arthritis (ReA) is defined as the development of sterile inflammatory arthritis classically following urogenital or gastrointestinal infections [1,2]. It is categorized as a form of spondyloarthritis, which can present as asymmetric oligoarthritis of the lower limb joints, sacroiliitis, enthesitis, or dactylitis [3]. According to Szamocki et al., there is a preponderance of ReA toward the male sex and those of Caucasian origin, between the ages of 20 and 40 years [4]. In the United States, the frequency of ReA is estimated at 3.5-5 patients per 100,000 [5].

Several bacteria have been linked to ReA, including *Chlamydia trachomatis*. Studies demonstrate that *C. trachomatis*, although identified as a common cause of ReA, only occurs in 1%-3% of infected persons with *Chlamydia*, out of an estimated annual incidence of three million infected people aged 15-44 years [1,6]. However, it is reasonable to assume that the rates of *Chlamydia* are underreported, and the following arthropathies are argued to be underdiagnosed. Although clinicians can treat the infection, the inflammation and consequence, that is, ReA, can require expert diagnosis and management. We detail a case of recurrent ReA induced by *C. trachomatis* infection in a young man requiring biologic disease-modifying antirheumatic drugs (DMARDs).

## Case presentation

A 32-year-old male patient with a past medical history significant for a traumatic meniscus tear of the left knee presented to the hospital for worsening joint pain over four weeks, which limited his ability to ambulate in July 2024. The pain started in the knee and spread to include the feet and back. Before presenting to the hospital, the patient was evaluated by Orthopedics, where he underwent an arthroscopy with 20 cc of yellow fluid drained, and Podiatry, where he received methylprednisolone injections with limited relief. The pain was associated with joint swelling, particularly in the knees, ankles, and feet. The physical examination yielded a normal general appearance, no oral ulcers or skin rashes, and his cardiopulmonary exam was also normal. The exam was significant for synovitis, primarily affecting the lower extremities, and dactylitis; there was no appreciated crepitus of the knees or limited range of motion. Laboratory findings were significant for an erythrocyte sedimentation rate (ESR) of 56 mm/h, a C-reactive protein of 67.4 mg/L, uric acid of 5.6 mg/dL, mildly elevated liver enzymes, and a positive HLA-B27. The laboratory results for Parvo B-19 were negative, and the patient had a positive Epstein-Barr virus (EBV) IgG without a positive IgM (Tables 1-3). An abdominal ultrasound was done for the elevated liver enzymes, which showed hepatosplenomegaly. An X-ray of the left knee showed mild joint space narrowing with no chondrocalcinosis. The patient was evaluated with a magnetic resonance imaging (MRI) of the sacroiliac joint, which was negative for signs of sacroiliitis. At this time, additional laboratory investigation yielded a positive *C. trachomatis* infection. The patient was diagnosed with ReA, and the patient was started on antibiotics and prednisone of 10 mg a day, which were uptitrated due to limited relief. The patient initially showed a response to prednisone 30 mg but experienced a recurrence of symptoms during the dose-tapering phase. Therefore, methotrexate, eight tablets weekly, and folic acid were added, which significantly relieved his symptoms and resulted in the normalization of his inflammatory markers. Notably, liver enzymes had also returned to normal levels.

**Table 1:** Complete blood count

Test name	Result	Reference value
White blood cells	14.0 thousand/µL	3.6-10.6 thousand/µL
Hemoglobin	14.8 g/dL	13.2-17.1 g/dL
Mean corpuscular volume	88.4 fL	80-100 fL
Platelet count	334 thousand/µL	140-400 thousand/µL

**Table 2:** Complete metabolic profile BUN: blood urea nitrogen; AST: aspartate aminotransferase; ALT: alanine aminotransferase

Test name	Result	Reference value
Sodium	142 mmol/L	135-146 mmol/L
Chloride	101 mmol/L	98-110 mmol/L
Potassium	5.0 mmol/L	3.5-5.3 mmol/L
Carbon dioxide	30.0 mmol/L	20-32 mmol/L
Calcium	9.9 mg/dL	8.6-10.3 mg/dL
BUN	14.0 mg/dL	7-25 mg/dL
Creatinine	0.8 mg/dL	0.6-1.25 mg/dL
AST	34 U/L	10-40 U/L
ALT	107 U/L	9-48 U/L
Total bilirubin	0.4 mg/dL	0.2-1.2 mg/dL

**Table 3:** Inflammatory markers and rheumatologic-related lab values

Test name	Result	Reference value
C-reactive protein	67.4 mg/L	<8.0 mg/L
Erythrocyte sedimentation rate	56 mm/hr	<15.0 mm/hr
Rheumatoid factor	<10 IU/mL	<14 IU/mL
Cyclic citrullinated peptide Ab	<16 units	-
Uric acid	5.6 mg/dL	4.0-8.0 mg/dL
HLA-B27	Positive	Negative

**Figure 1:**
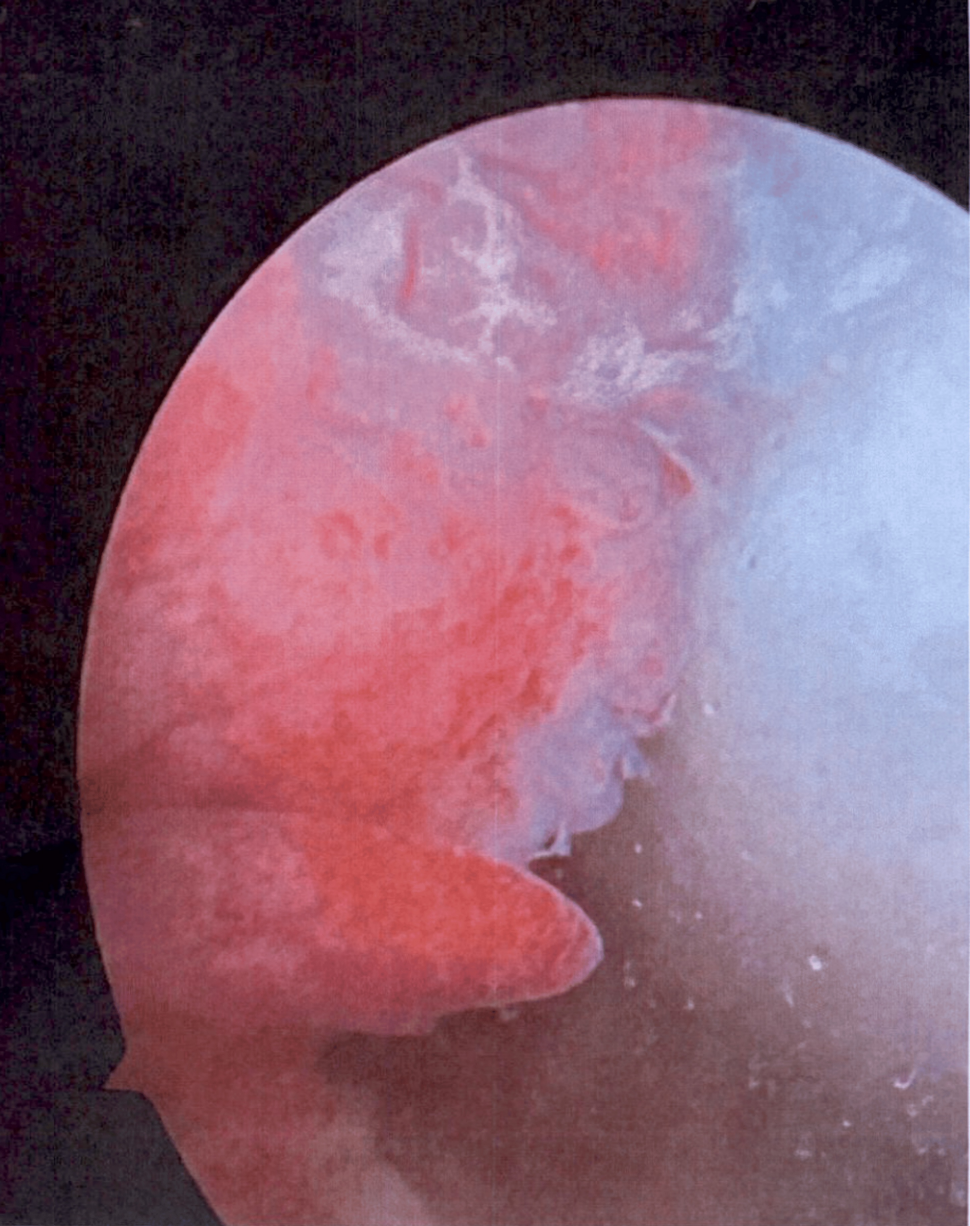
Arthroscopy of the left knee: 2 × 2 full-thickness cartilage lesion at the level of the medial femoral condyle, consistent with extensive synovitis

The patient continued to follow up as an outpatient following discharge from the hospital. His pain was managed with a slow taper of prednisone and methotrexate with folic acid. In January 2025, he presented as a follow-up to the outpatient clinic with an exacerbation of ReA. The symptoms reappeared following left knee surgery for the aforementioned meniscal tear and a subsequent bout of food poisoning. Following the food poisoning, the patient experienced diarrhea, loss of appetite, night sweats, dactylitis of the toes, and significant joint pain affecting the knees, lower back, neck, and feet. Laboratory studies were repeated and were significant for an ESR of 60 mm/hr. Preoperatively, the patient underwent arthroscopy, which revealed large, grade 4 lesions, the most severe cartilage damage, as a result of chronic synovitis (Figure 1). Furthermore, a biopsy taken from the knee surgery confirmed chronic synovitis, aligning with the diagnosis of ReA. The patient was started on a low dose of prednisone with minimal improvement, and only experienced relief at a dose upward of 30 mg. The patient intended to have another surgery on his right knee in the following two months. Treatment options were discussed, and the patient agreed to initiate treatment with adalimumab with the continuation of the prednisone taper. The patient has regained a full range of motion, and his symptoms remain controlled with methotrexate and prednisone until adalimumab therapy was initiated. After the initiation of adalimumab, the patient has remained in remission and was successfully tapered off prednisone and is now being tapered off methotrexate.

## Discussion

This case demonstrates a persistent ReA that was initially triggered by a *C. trachomatis* infection in a young man and details how medical management was tailored to his condition. As stated, the prevalence of ReA is 3.5-5 patients per 100,000 [7]. *C. trachomatis*-induced ReA is more prevalent in men and typically occurs within 1-4 weeks after the initial infection [1]. The development of ReA is closely linked with infection, genetic factors, and immune abnormalities. Specifically, the presence of the HLA-B27 allele is associated with an increased risk and more severe or chronic disease progression [8]. After a local bacterial infection, the bacterial antigens or peptides are transported from the primary site into the synovial membrane by antigen-presenting cells (APCs), activating T-lymphocytes against bacterial antigens or peptides. This activation triggers a surge of inflammatory cytokines, resulting in synovial inflammation [9]. When *C. trachomatis* infects certain host cells, such as monocytes, the body releases pro-inflammatory cytokines, which induce persistent *C. trachomatis* infection. This is mainly because *C. trachomatis* in some way inhibits the combination of phagosomes and lysosomes, resulting in a state whereby *C. trachomatis* lives inside the cells [10].

The diagnosis of ReA is clinical and is based on the physical presentation, evidence of a preceding infection, and laboratory findings [5]. ReA manifests as asymmetric oligoarthritis, often predominant in the lower limbs, with a history of a preceding or ongoing infection. The classic triad of ReA includes arthritis, urethritis, and conjunctivitis; however, this is seen in a minority of patients [11]. Diagnosis is further supported by elevated inflammatory markers (ESR or C-reactive protein). Diagnosis is also supported by synovial fluid analysis, which typically shows sterile inflammation and assists in excluding other causes of joint inflammation, such as crystal-induced or septic arthritis. Lastly, regarding laboratory investigation, testing for the HLA-B27 allele is linked to the increased recurrence and chronicity of ReA. However, allele testing is not required to confirm the working diagnosis, nor does a negative result exclude ReA [12]. In addition to laboratory findings, imaging can be used to assess the extent of joint involvement and monitor disease progression. Plain radiographs, ultrasound, and MRI are not necessary to confirm the diagnosis of ReA, but distinct findings on each of these imaging modalities can contribute to the accurate diagnosis and treatment of the disease. Often normal in early disease, plain radiographs may show asymmetric joint space narrowing, or periosteal reactions, specifically at the Achilles tendon or plantar fascia if these are the affected areas [13]. Ultrasound helps detect enthesitis, tenosynovitis, or joint effusion and offers a non-invasive method for evaluating soft tissue inflammation and assessing the role of joint aspiration. Finally, MRI provides superior soft tissue resolution and can detect early sacroiliitis and bone marrow edema to help support a diagnosis of ReA [14]. There is no widely accepted diagnostic criterion or score that clinicians can use to diagnose ReA, so diagnosis is dependent on the clinical manifestation of the ReA, including the asymmetric oligoarthritis, preceding genitourinary or gastrointestinal features, and supportive laboratory and imaging findings.

The treatment for ReA depends on the phase of the disease and whether it is an acute, persistent, or chronic presentation. Generally, the treatment options for ReA include non-steroidal anti-inflammatory drugs (NSAIDs), glucocorticoids, DMARDs, and tumor necrosis factor (TNF)-alpha inhibitors [15]. For the acute phase of treatment, NSAIDs are the first-line treatment of symptomatic relief, followed by corticosteroids. Typically, systemic steroids are reserved for persistent cases. For chronic ReA, defined as lasting for more than six months, patients can initiate DMARD treatment. In the instance of refractory cases or axial involvement, there is limited data that support the initiation of TNF-alpha inhibitor therapy [16]. In addition to medication therapy, patients should be supported with conservative measures like physical therapy. Finally, there is new research investigating the role of a prolonged course of antibiotics in the treatment of ReA. Beyond the treatment of the triggering disease, a randomized control trial by Carter et al. showed that a six-month course of combination antibiotics achieved greater rates of remission among patients [17]. The use of long-term antibiotics has yet to be further investigated, but it would serve as an additional treatment option for patients.

Our patient received antibiotic treatment for the underlying *C. trachomatis* infection as well as immunomodulatory therapy for symptomatic joint disease. Despite the initial treatment for *C. trachomatis*-induced ReA, our patient on follow-up had subsequent flare-ups following aggravating infections. Following this patient over several months also shows the relapsing and remitting nature of his disease and how therapy was escalated with the subsequent flare-ups. This patient also supports the theory that HLA-B27-positive patients have increased susceptibility to recurrence and the development of chronic ReA. In a Finnish-Danish study, the presence of a positive HLA-B27 antigen in the assessment of ReA triggered by non-gonococcal genitourinary infection was related to frequent chronic back pain, and follow-up studies showed radiological evidence of the progression of sacroiliitis in up to 30% of patients. Additionally, it has been reported that although most patients achieve clinical remission within six months, chronic inflammation with periods of relapsed symptomatology has also been linked to *C. trachomatis*-induced ReA [6]. 

As previously stated, *C. trachomatis*-induced ReA is often underdiagnosed, and those who develop ReA either achieve remission without medical attention or are misdiagnosed. Typically, these patients are diagnosed with other rheumatologic conditions, most often seronegative rheumatoid arthritis or psoriatic arthritis without psoriasis [1]. Epidemiologic data support that asymptomatic presentation of *C. trachomatis* presents a hurdle to an accurate diagnosis of induced ReA versus other immunologic pathologies [1,6]. It is important to consider venereal disease as a trigger for ReA in at-risk populations and recognize that the inflammation and symptoms may result in chronic arthritis despite adequate and timely antibiotic treatment. Timely recognition of ReA, particularly the sexually transmitted form, has both individual and public health implications. It allows for appropriate antibiotic therapy, reduces the risk of chronic joint sequelae, and prompts partner notification and treatment to prevent reinfection and transmission [17]. Furthermore, in the instances of persistent ReA, it is essential to treat the symptomatology of the patient and gain a better understanding of the long-term risks associated with sexually transmitted infection (STI)-triggered spondyloarthropathies.

## Conclusions

There are several hurdles to the underdiagnosis of *C. trachomatis*-induced ReA, which potentiate decreased awareness and improper treatment. Our case demonstrates the diagnosis and provides the opportunity to follow a case of *C. trachomatis*-induced ReA and how treatment in the case of a persistent case should be escalated to properly address the patient's symptomatology. Furthermore, this case provides more insight into the long-term effects and prognosis of this disease process, which remain largely understudied and would benefit from further investigative studies. Lastly, our case highlights a common condition for clinicians to include in their differential diagnoses in at-risk populations.
